# Changes Induced by Pressure Processing on Immunoreactive Proteins of Tree Nuts

**DOI:** 10.3390/molecules25040954

**Published:** 2020-02-20

**Authors:** Carmen Cuadrado, Africa Sanchiz, Fatima Vicente, Isabel Ballesteros, Rosario Linacero

**Affiliations:** 1 Departamento de Tecnología de Alimentos, SGIT-INIA, Ctra. La Coruña Km. 7.5, 28040 Madrid, Spain; africa.sanchiz@gmail.com (A.S.); fatima.vicente.martin@gmail.com (F.V.); 2 Facultad de Ingeniería y Ciencias Aplicadas, Grupo de Investigación en Biotecnología Aplicada a Biomedicina (BIOMED), Universidad de Las Américas, 72819 Quito, Ecuador; isabelbare@gmail.com; 3 Departamento de Genética, Fisiología y Microbiología Facultad de Biología, Universidad Complutense de Madrid, 28040 Madrid, Spain; charolin@ucm.es

**Keywords:** pistachio, cashew, peanut, hazelnut, almond, chestnut, allergens, pressure processing, thermal processing

## Abstract

Tree nuts confer many health benefits due to their high content of vitamins and antioxidants, and they are increasingly consumed in the last few years. Food processing is an important industrial tool to modify allergenic properties of foods, in addition to ensuring safety and enhancing organoleptic characteristics. The effect of high pressure, without and with heating, on SDS-PAGE and immunodetection profile of potential allergenic proteins (anti-11S, anti-2S and anti-LTP) of pistachio, cashew, peanut, hazelnut, almond, and chestnut was investigated. Processing based on heat and/or pressure and ultra-high pressure (HHP, 300–600 MPa) without heating was applied. After treating the six tree nuts with pressure combined with heat, a progressive diminution of proteins with potential allergenic properties was observed. Moreover, some tree nuts proteins (pistachio, cashew, and peanut) seemed to be more resistant to technological processing than others (hazelnut and chestnut). High pressure combined with heating processing markedly reduce tree nut allergenic potential as the pressure and treatment time increases. HHP do not alter hazelnut and almond immunoreactivity.

## 1. Introduction

Food allergy affects to 1–3% in the general population, and it reaches up to 8% in children. Currently, food allergy is the main cause of anaphylactic reactions treated in hospital emergency departments in Western countries. It has been estimated that food allergy causes approximately 30,000 anaphylactic reactions and 2000 hospitalizations annually in the U.S. [1]. In the European Union, tree nuts are included in the list with the 14 most allergenic ingredients, according with this regulation, and its presence must be indicated in foods. Tree nuts are, after fruits, the most implied allergens (26%) in allergic reactions in Spain [2]. Despite these facts, tree nuts consumption is on the rise because of its recognized health benefits. Tree nuts are valuable foods rich in proteins, minerals, vitamins, antioxidants, and a considerable high content of unsaturated fatty acids, and their global production is increasing in the last years [3].

Most nut allergens are seed storage proteins such as vicilins (7S globulin subunits composed of approximately 50–60 kDa), legumins (11–13S globulin subunits composed of acidic peptides of 30–40 kDa and basic 15–20 kDa) and 2S albumin (15 kDa) [4]. Other allergens of nuts with known biological function, such as lipid transfer proteins (LTP), profilins, and proteins homologous to pathogenesis-related (PR) proteins are considered panallergens because they contribute to the allergenicity of a large group of pollen, nuts, seeds, fruits, and other plants [5]. A common feature of the nut allergenic proteins is their resistance to proteolysis and denaturation [6].

Among nuts, cashew (*Anacardium occidentale*) allergy is a significant health problem and the second most allergic nut in the U.S. [7]. Clinical manifestations of allergy to cashew often are severe anaphylactic reactions, and even more severe than with peanuts [7]. Three allergenic proteins of cashew have been identified and characterized: Ana o 1 (7S vicilin, 50 kDa) [8], Ana o 2 (11S legumin, 55 kDa) [9], and Ana o 3 (2S albumin, 14 kDa) [10]. Pistachio (*Pistacia vera*) is another well characterized nut for its allergenic potential and cross-reactivity with cashew and mango [11]. Similar to cashew, the 5 pistachio major allergens correspond to seed storage proteins: two 11S legumins (Pis v 2 and 5, 32 and 36 kDa), a 2S albumin (Pis v 1, 7 kDa), one 7S vicilin (Pis v 3, 55 kDa), and a superoxide dismutase (Pis v 4, 25.7 kDa) [12,13,14]. Peanut allergy is one of the most common IgE-mediated reactivity to food because of its severity and lifelong persistence [15]. Considerable effort has been spent in characterizing peanut allergens and eleven allergenic proteins have been identified until now (Ara h 1-Ara h 11). The major peanut allergens, Ara h 1 (65 kDa, vicilin) and Ara h 2 (17 kDa, conglutin), are recognized by 70–90% of sensitized subjects [16] and Ara h 3 (11 S legumin) has been considered to play a lesser allergenic role [17]. Food allergies to hazelnut and almond represent an important health problem in industrialized countries because of their high prevalence and severity [18]. Several hazelnut allergens are well characterized being Cor a 1 (18 KDa, Bet v 1 family) the major one. Other allergenic proteins are Cor a 2 (profilin), Cor a 8 (lipid transfer protein, LPT), Cor a 9 (11S globulin), Cor a 11 (vicilin-like protein), and Cor a 12, Cor a 13, and Cor a 14 belonging to the 2S albumins [19]. So far, eight allergenic proteins have been described in almond: Pru du 1 (PR-10 protein), Pru du 2 (thaumatin-like protein), Pru du 2S albumin, Pru du 3 (lipid transfer protein), Pru du 4 (profilin), Pru du 5 (60S ribosomal protein), Pru du 6 (11S-legumin), and Pru du γ-conglutin (7S vicilin) [20]. Of these eight allergens, Pru du 3, Pru du 4, Pru du 5, and Pru du 6 are recognized as such and included in the WHO−IUIS allergen list. Allergy to chestnuts has been widely reported in the latex-fruit syndrome [21,22]. However, few studies address actual allergy to chestnuts in patients reacting primarily to this food. According to WHO-IUIS list three food allergens have been identified until the date in this tree nut, all of them from the plant defense system: Cas 5, a class I chitinase [23], Cas s 8, a non-specific type I lipid transport protein (LTP, lipid transfer protein) [24] and Cas s 9, a cytosolic class I small heat shock protein [25]. Additionally, a thaumatin like protein, Cas s TLP, has been described as chestnut food allergen [26].

Food processing, that is used in food industry for several applications, can alter the structure, function, and properties of proteins, and thereby also modify the IgE reactivity of allergens, in such a way that it has been proposed as a method to obtain food with altered allergenicity. Some daily processing methods have been shown to be effective in decreasing the content of specific allergens in certain foods, which may open a future path for hypoallergenic food development or pave the way to the use of specifically processed foods for tolerance induction. Other treatments, however, have the capacity to increase the allergenicity of certain foods [27]. Food processing, particularly heat treatment, may reduce the allergenicity of some foods, so it could be useful for the control of their allergenic risk [28]. Thermal treatment changes the structure of proteins, their immunogenic potential, and, therefore, the overall allergenicity of the food. These effects depend on the temperature, type, and duration of the treatment, as well as of the intrinsic characteristics of the protein and of the physicochemical conditions of its microenvironment [29,30]. There are no general rules about the effect of processing on the allergenicity and hence, it has the ability to generate new allergenic epitopes (neoallergens) as well as to abolish the existing reactive epitopes [31,32,33]. Several studies have evaluated the structural changes induced by treatments such as boiling, microwave heating and pressure-cooking and their effects on legumes and nuts allergenicity, showing that processing based on pressure and heat seems to have an important impact on reducing in vitro IgE-binding capacity [31,32,34,35,36]. High-hydrostatic pressure (HHP) is considered an emerging processing technology used to develop novel and high-quality foods. This novel-processing technique even renders harmless foods, which would be of considerable benefit to consumers. HHP treatment of foods can be used to create new products (new texture or taste) or to obtain analog products with minimal effect on flavor, color, and nutritional value and without any thermal degradation. It is well established that higher pressure has a disruptive effect on the tertiary and quaternary structure of most globular proteins, with relatively little influence on the secondary structure [37,38]. Therefore, higher hydrostatic pressure can unfold proteins. The typical pressure needed for the unfolding is around 500 MPa but it varies from protein to protein, in the range from 100 MPa to 1 GPa or reaching even higher pressures in special cases. The effect of HHP on immunoreactive proteins is being currently investigated through changes in protein structure [39]. Such effects have been studied in beef [40,41], apple [37,38], celery [37], and in nuts such as peanut [38]. However, there is scarce information on the effects of such food processing techniques on hazelnut and almond immunoreactive proteins.

Our aim is to study the effect of high pressure, without and with heating, on SDS-PAGE and immunodetection profile of potential allergenic proteins (anti-11S, anti-2S and anti-LTP) of pistachio, cashew, peanut, hazelnut, almond, and chestnut.

## 2. Results and Discussion

### 2.1. Electrophoretic Pattern and Immunodetection Assays of Processed Cashew

**Figure 1:**
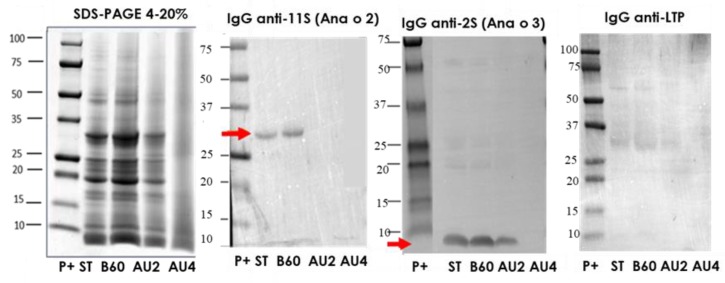
SDS-PAGE and IgG anti-11S, anti-2S, and anti-LTP immunoblots analysis of control (ST), boiled for 60 min (B60) and autoclaved at 121 °C for 30 min (AU2) and at 138 °C for 30 min (AU4) cashew samples. Molecular weight marker was Precision Plus (P+).

The characterization of the electrophoretic profile of the soluble protein extracts from untreated and thermal-treated cashew samples is shown in Figure 1. The results showed that the treatments of boiling during 60 min had no major effects on the IgG-binding proteins from cashew. However, cashew subjected to heat and pressure treatments (autoclave) showed less distinctive stained bands in SDS-PAGE with an increased protein fragmentation that went along with a reduction in IgG-reactive bands.

We additionally studied the electrophoretic and IgE-binding patterns of total protein extracts from cashew, which were obtained by direct solubilization of untreated and treated cashew flours in the SDS sample buffer as described in Materials and Methods (Supplementary material. Figure S1). A band around 13 kDa probably corresponding to the 2S albumin Ana o 3 [10] was especially immunoreactive. The IgE reactive bands were strongly reduced in the samples treated with heat and pressure, but possible to detect even in the sample AU at 138 °C, 15 min.

In the Western blot with IgG anti-11S, anti-2S, and anti-LTP (Figure 1), arrows indicate the protein bands detected (Ana o 2 and Ana o 3). 11S is not affected by boiling but is eliminated by autoclave. The albumin 2S is resistant after autoclave treatment at 121 °C but is not detected after autoclave at 138 °C. No immunoreactive bands were detected when IgG anti-LTP was applied. In the IgE Western blot using a pool of sera from allergic patients, (Supplementary material. Figure S1), apart from other bands, we saw that a major band around 15kDa was persistent and detectable until 121 °C 30 min.

### 2.2. Electrophoretic Pattern and Immunodetection Assays of Pistachio Samples

**Figure 2:**
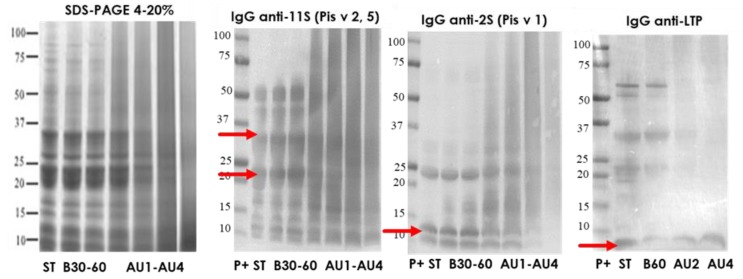
SDS-PAGE and IgG anti-11S, anti-2S, and anti-LTP immunoblots analysis of control (ST), boiled 30 and 60 min (B30, B60) and autoclaved at 121°C 15 and 30 min (AU1, AU2) and 138 °C, 15 and 30 min (AU3, AU4) pistachio samples. Molecular weight marker was Precision Plus (P+).

The protein profile, visualized by SDS-PAGE, of pistachio protein extract from untreated and boiling treated samples was very similar (Figure 2). A low number of bands was reduced after the softest heat/pressure treatment (AU 121 °C, 15 min) and the rest of heat/pressure treatments (AU 121 °C, 30 min, AU 138 °C, 15 min and AU 138 °C, 30 min) provoked a smear due to the degradation, rich in low molecular weight proteins. The most influenced processing effect was obtained after harsh heat/pressure conditions (138 °C for 30 min) (Figure 2). Western blot with IgG anti-11S, anti-2S, and anti LTP is also analyzed. Arrows indicate the protein band detected (Pis v 2/5 and Pis v 1). There is an important reduction of 11S and 2S detection after autoclave treatment at 138 °C. However, LTP was even detected after autoclaving at 138 °C, 30 min. IgE reactivity reduction was of 73% after boiling treatment in pistachio proteins and the lowest detection was at the hardest autoclave conditions (Supplementary material Figure S2).

### 2.3. Electrophoretic Pattern and Immunodetection Assays of Processed Peanut Samples

**Figure 3:**
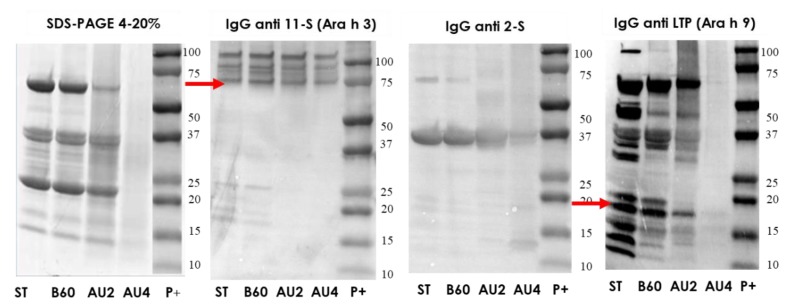
SDS-PAGE and IgG anti-11S, anti-2S and anti-LTP immunoblots analysis of control (ST), boiled 60 min (B60) and autoclaved at 121°C for 30 min (AU2) and 138 °C for 30 min (AU4) peanut samples. Molecular weight marker was Precision Plus (P+).

The characterization of the electrophoretic profile of the soluble protein extracts from untreated and thermal-treated cashew samples is shown in Figure 3. The results showed that the treatments of boiling during 60 min had no major effects on the IgG-binding proteins from peanut. However, peanut subjected to heat and pressure treatments (autoclave) showed less distinctive stained bands in SDS-PAGE with an increased protein fragmentation that went along with a reduction in IgG-reactive bands. In the Western blot with IgG anti-11S, anti-2S, and anti-LTP, arrows indicate the protein bands detected (Ara h 3 and Ara h 9). 11S is not affected by boiling or autoclave. The albumin 2S is resistant after autoclave treatment at 121 °C and is weakly detected after autoclave at 138 °C. Some immunoreactive bands were detected when IgG anti-LTP was applied. The putative Ara h 9 band was not detected after autoclave at 138 °C, 30 min.

### 2.4. Electrophoretic Pattern and Immunodetection Assays of Processed Hazelnut Samples

**Figure 4:**
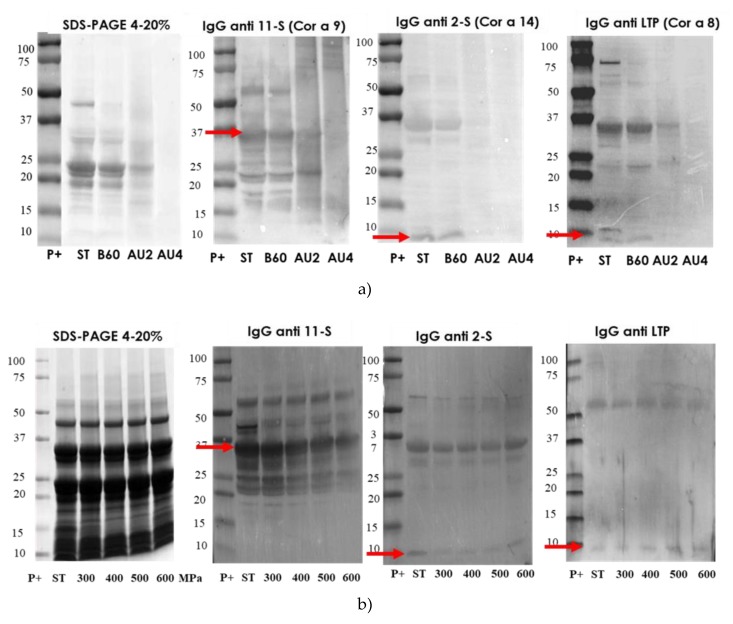
(**a**) SDS-PAGE and IgG anti-11S, anti-2S, and anti-LTP immunoblots analysis of control (ST), boiled 60 min (B60) and autoclaved at 121°C at 30 min (AU2) and 138 °C 30 min (AU4) hazelnut samples. (**b**) SDS-PAGE and IgG immunoblot analysis of control and high-hydrostatic pressure (HHP) 300 to 600 MPa (300, 400, 500, 600) processed hazelnut samples Molecular weight marker was Precision Plus (P+).

The protein profile by SDS-PAGE, of hazelnut protein extract from untreated and boiling treated samples was very similar (Figure 4a). A number of bands was reduced after heat/pressure treatment at 121 °C, 30 min) and the highest diminution was obtained after harsh heat/pressure conditions (138 °C for 30 min) (Figure 4a). Western blot with IgG anti-11S, anti-2S, and anti LTP is also analyzed and arrows indicate the protein band detected (Cor a 9, Cor a 14 and Cor a 8). There is an important reduction of 11S, 2S, and LTP detection after autoclave treatment at 138 °C. IgE reactivity was reduced following autoclave treatments (Supplementary material Figure S3).

### 2.5. Electrophoretic Pattern and Immunodetection Assays of Processed Almond Samples

**Figure 5:**
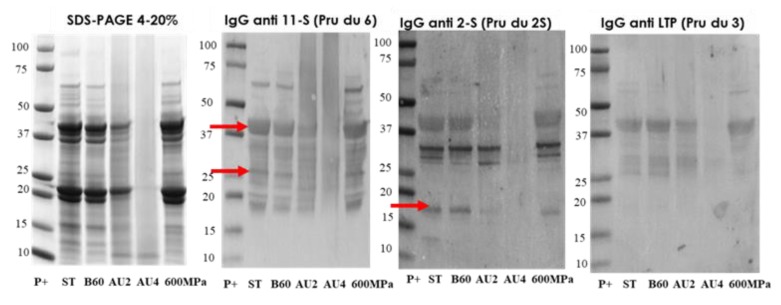
SDS-PAGE and IgG anti-11S, anti-2S, and anti-LTP immunoblots analysis of control (ST), boiled 60 min (B60) and autoclaved at 121°C at 30 min (AU2) and 138 °C 30 min (AU4) and HHP treated (600 MPa) almond samples. Molecular weight marker was Precision Plus (P+).

The electrophoretic profile of the soluble protein extracts from untreated and processed almond samples is shown in Figure 5. Western blot with IgG anti-11S, anti-2S, and anti LTP is also analyzed and arrows indicate the protein band detected (Pru du 6, Pru du 2S, and Pru du 3). The results showed that the treatments of boiling during 60 min had no major effects on the IgG-binding proteins from almond. However, almond subjected to heat and pressure treatments (autoclave) showed less distinctive stained bands in SDS-PAGE with an increased protein fragmentation that went along with a reduction in IgG-reactive bands. The electrophoretic migration patterns of high-pressure treated flour almond proteins were similar to the control almond as well as the immunodetection IgG profile.

### 2.6. Electrophoretic Pattern and Immunodetection Assays of Processed Chestnut Samples

**Figure 6:**
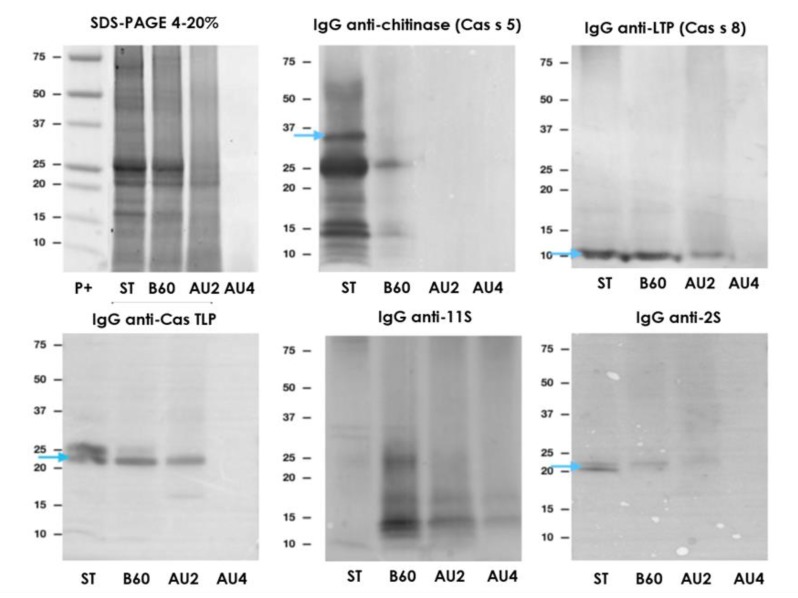
SDS-PAGE and IgG anti-chitinase, anti-lipid transfer protein (anti-LTP), anti TLP, anti-11S, and anti-2S immunoblots analysis of control (ST), boiled 60 min (B60) and autoclaved at 121°C at 30 min (AU2) and 138 °C 30 min (AU4) chestnut samples. Molecular weight marker was Precision Plus (P+).

The immunodetection results in chestnut are summarized in Figure 6. SDS-PAGE showed a similar profile between untreated and boiled samples, although the immunoblot is slightly variable. Resistant bands around 25 and 15 kDa were not detected after autoclave. IgE reactivity reduction was of 76% after boiling treatment of chestnut (Supplementary material Figure S5). Thermal treatments (boiling and autoclave) diminished protein bands’ number, especially after pressure at 138 °C for 30 min, and modified protein solubility. Detection was possible with all antibodies after boiling for 60 min (Figure 6). After autoclaving, there is a strong reduction in the immunoreactive proteins’ detection. After Western blot with anti-11S, the detection was possible even at autoclave harshest conditions (Figure 6).

In this study, the influence of moist thermal treatments on the imunoreactivity of cashew, pistachio, peanut, hazelnut, almond, and chestnut has been analyzed. The results in Figure 1, Figure 2, Figure 3, Figure 4, Figure 5 and Figure 6 showed that heat and pressure treatment at the harshest conditions considered (138 °C, 2.56 atm, 30 min) produced a higher decrease of the IgG-binding capacity of the six tree nuts proteins than boiling without pressure or soft conditions of heat and pressure. Interestingly, although the treatments of heat and pressure seemed to affect cashew to a greater extent than pistachio allergens (Figure 1 and Figure 2) both besides peanut proteins (Figure 3) seemed to be more resistant to technological processing than others (hazelnut, almond and chestnut) (Figure 4, Figure 5 and Figure 6). In the specific case of cashew and pistachio, the effects of thermal processing on their allergenic properties have been addressed by a limited number of studies that used assays that evaluated IgE-binding in Western blot or ELISA [42]. In our study, we have applied thermal treatments to cashew and pistachio that included not only boiling without pressure and heat/pressure treatments at soft conditions, but also harsher conditions of heat and pressure (138 °C, 2.56 atm, during 15 and 30 min), which turned to be the most efficient treatment to decrease the allergenic properties of both tree nuts considered in our study. The harshest conditions of heat and pressure applied in our study produced a degradation of all the tree nuts studied (cashew, pistachio, peaenut, hazelnut, almond, and chestnut), with an increased protein fragmentation seen as an intense smear in the low molecular weight area in the SDS-PAGE. Such alteration in the electrophoretic and IgG-binding patterns after heat and pressure treatments cannot be explained by a potential loss of solubility of proteins due to the thermal treatments, since the experiments carried out with strong conditions of protein solubilization (flours directly solubilized in SDS-PAGE sample buffer) showed the same pattern of protein degradation for heat and pressure treated samples. The degradation of proteins after harsh heat/pressure treatments obtained in our study reminds to the degradation produced by some enzymatic treatments [33,43,44].

## 3. Materials and Methods

### 3.1. Plant Material and Processing

**Figure 7:**
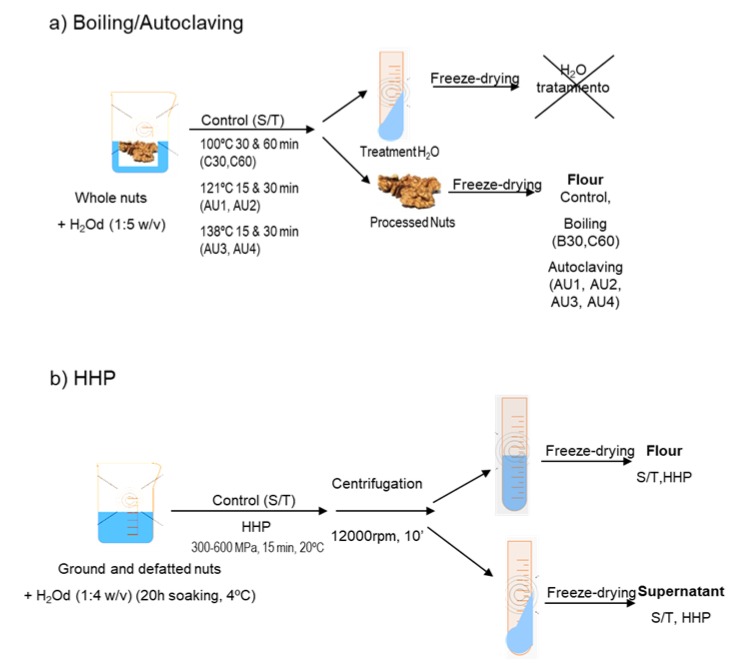
Scheme of (**a**) boiling and autoclave treatments and (**b**) high hydrostatic pressure (HHP) treatment of tree nut samples.

Cashew (*Anacardium occidentale*, type 320) and peanut (*Arachys hypogaea*, Virginia) obtained from Productos Manzanares S.L. (Spain) and pistachio (*Pistachia vera* Kerman), hazelnut (Corylis avellana, Negreta), and almond (Prunus dulcis, Marcona) from the Germoplasm Bank of Institut de Recerca i Tecnologia Agroalimentaries (IRTA-Mas de Bover, Tarragona, Spain) were used for this study. An additional commercial variety of chestnut (*Castanea sativa*), Miquelenca, was purchased to Cooperativa Secallona (Viladrau, Gerona, Spain). Whole nuts seeds were immersed in distilled water (1:5 w/v) and boiled (100 °C, 30 and 60 min, (B30 and B60)) or autoclaved using an autoclave Compact 40 Benchtop (Priorclave, London, UK) at 121 °C, 120 kPa for 15 and 30 min (AU1 and AU2) and at 138 °C, 256 kPa, for 15 and 30 min (AU3 and AU4) (Figure 7a). Untreated, boiled and autoclaved nut seeds were ground and defatted with n-hexane (34 mL/g of flour) for 4 h, shaken, and air-dried after filtration of the n-hexane. Defatted flour from untreated samples was the control for boiled and autoclaved samples. Hazelnuts and almonds were ground and defatted with n-hexane (34 mL/g of flour) for 4 h and air-dried after filtration of the n-hexane. High-pressure experimental conditions were carried out according to Omi et al. [45] and Kato et al. [46]. Hazelnut and almond defatted flours were dissolved in distilled water (1:4 w/v) 20 h before HHP treatment (Figure 7b). The flours were subjected to HHP, using pressures of 300, 400, 500, and 600 MPa for 15 min in a multivessel high-pressure equipment (HHP, ACB, France) at 20 °C (Figure 6).The nitrogen contents of the samples were determined by LECO analysis according to standard procedures based on Dumas method [47]. The total protein content was calculated as N x 5.3 [47]. The analyses were carried out in duplicate.

### 3.2. Protein Electrophoresis and Immunoblot Experiments

Protein electrophoresis of the defatted flours was carried out as previously described [33]. Defatted flours from untreated and treated samples were dissolved in standard electrophoresis sample buffer. The same amount of protein (20 μg) calculated from LECO analysis from each sample was electrophoresed in 4–20%. Proteins were visualized with Coomassie Brilliant Blue (Bio-Rad, Hercules, CA, USA). For Western blot, proteins were transferred to polyvinylidene difluoride (PVDF) membranes ((Millipore Corp., Bedford, MA, USA). Blocking was carried out for 1 h at room temperature in PBS plus 0.5% Tween-20 (PBST) containing 3% milk (blocking solution). IgG mouse anti-11S (dilution 1:10,000), anti-2S (1:25,000), anti-chitinase (1:200), anti TLP (1:5000), and anti-LTP (1:500) were diluted in blocking solution and incubated with the PVDF membranes for 1 h. Membranes were washed and then treated with alkaline phosphatase (AP) conjugated goat anti mouse antibody (1:5000) (Sigma, Saint Louis, MO, USA) diluted in blocking solution. Detection was achieved by means of BCIP/NBT substrate (Sigma, Saint Louis, MO, USA). The signal was measured using ChemiDoc (Bio-Rad, Hercules, CA, USA). For IgE Western blot, proteins were transferred to a polyvinylidene difluoride (PVDF) membrane and the membranes were incubated overnight at 4 °C with pool sera from 6 patients with tree nuts, washed and then treated with Horseradish peroxidase (HRP) conjugated mouse anti-human IgE (1:10,000 dilution for 30 min at RT) (Sigma, Saint Louis, MO, USA). Detection of IgE-binding proteins was achieved by means of enhanced chemiluminescence, according to the manufacturer’s instructions (Thermo Scientific, Waltham, MA, USA). The signal was measured using CCD camera system of ChemiDoc (Bio-Rad, Hercules, CA, USA).

## 4. Conclusions

Thermal and pressure treatment (autoclaving) was able to decrease IgE binding properties of pistachio, cashew, peanut, hazelnut, almond, and chestnut. Autoclave 138 °C (256Kpa) for 30 min showed the most relevant reduction in IgG reactivity in all the tree nuts. After autoclave at 138 °C for 30 min, IgG immunodetection of Pis v1, Pis v 2, Pis v 5, Ana o 2, Ana o 3, Ara h 9, Cor a 9, Cor a 14, Cor a 8, Pru du 6, Pru du 2S, Pru du 3, Cas s 5, Cas s 8, and Cas s TLP is strongly diminished. More studies are necessary in order to obtain more conclusive results, such as basophil activation assay with human cells.

## Supplementary Materials

The following are available online. Figure S1: Immunodetection in cashew, Figure S2. Immunodetection in PISTACHIO, Figure S3. Western blot in hazelnut (3 patients), Figure S4. Western blot in almond (pool 4 patients), Figure S5. Immunodetection in Chesnut.
