# Supplementary material for: Changes Induced by Pressure Processing on Immunoreactive Proteins of Tree Nuts
Source: Molecules. 2020 Feb 20;25(4):954. doi: 10.3390/molecules25040954 (PMC7070680; doi:10.3390/molecules25040954)
Supplement: Supplementary file 1 [file molecules-25-00954-s001.pdf]

FIGURE S1. IMMUNODETECTION IN CASHEW

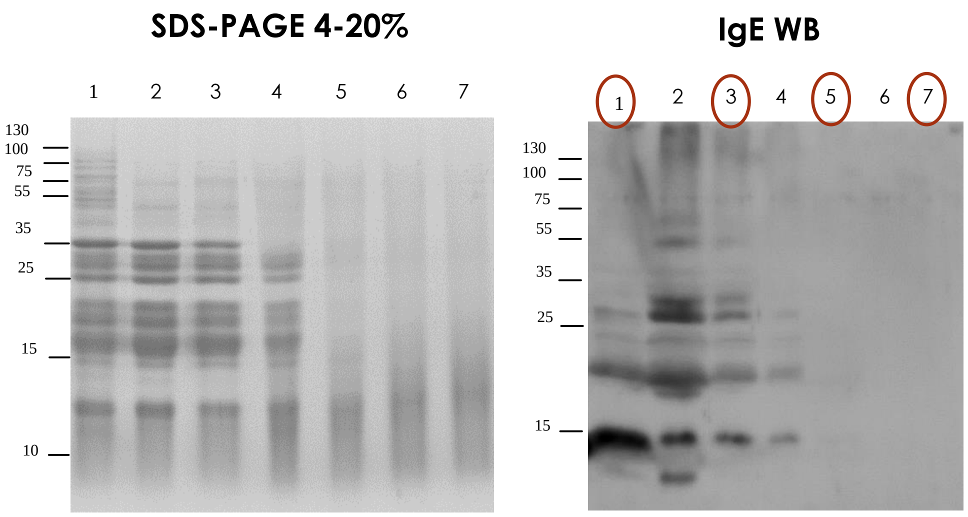

1- Untreated
2- Boiled 30 min
3- Boiled 60 min
4- Auto 121°C 15 min
5- Auto 121°C 30 min
6- Auto 138°C 15 min
7- Auto 138°C 30 min

FIGURE S2. IMMUNODETECTION IN PISTACHIO

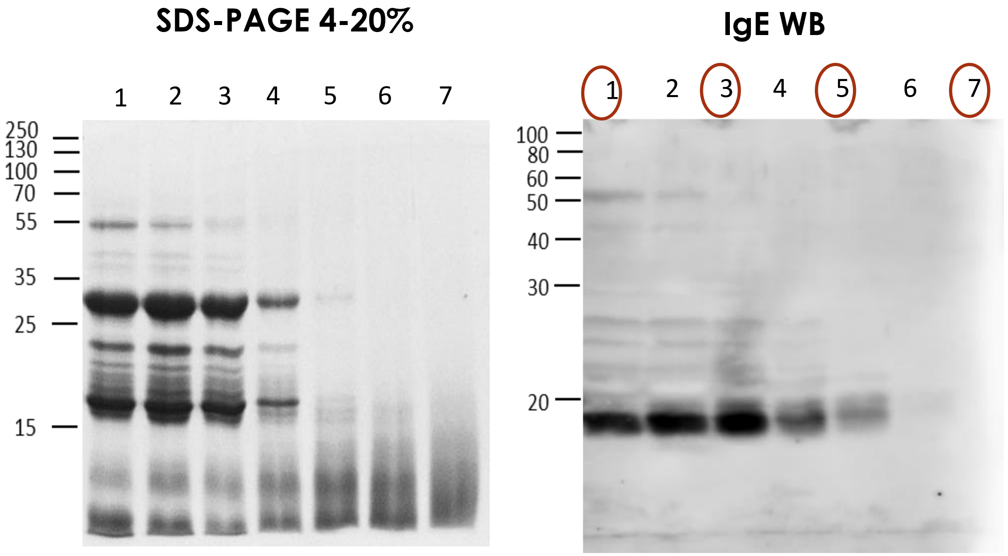

1- Untreated
2- Boiled 30 min
3- Boiled 60 min
4- Auto 121°C 15 min
5- Auto 121°C 30 min
6- Auto 138°C 15 min
7- Auto 138°C 30 min

Figure S3. Western blot de hazelnut (3 patients)

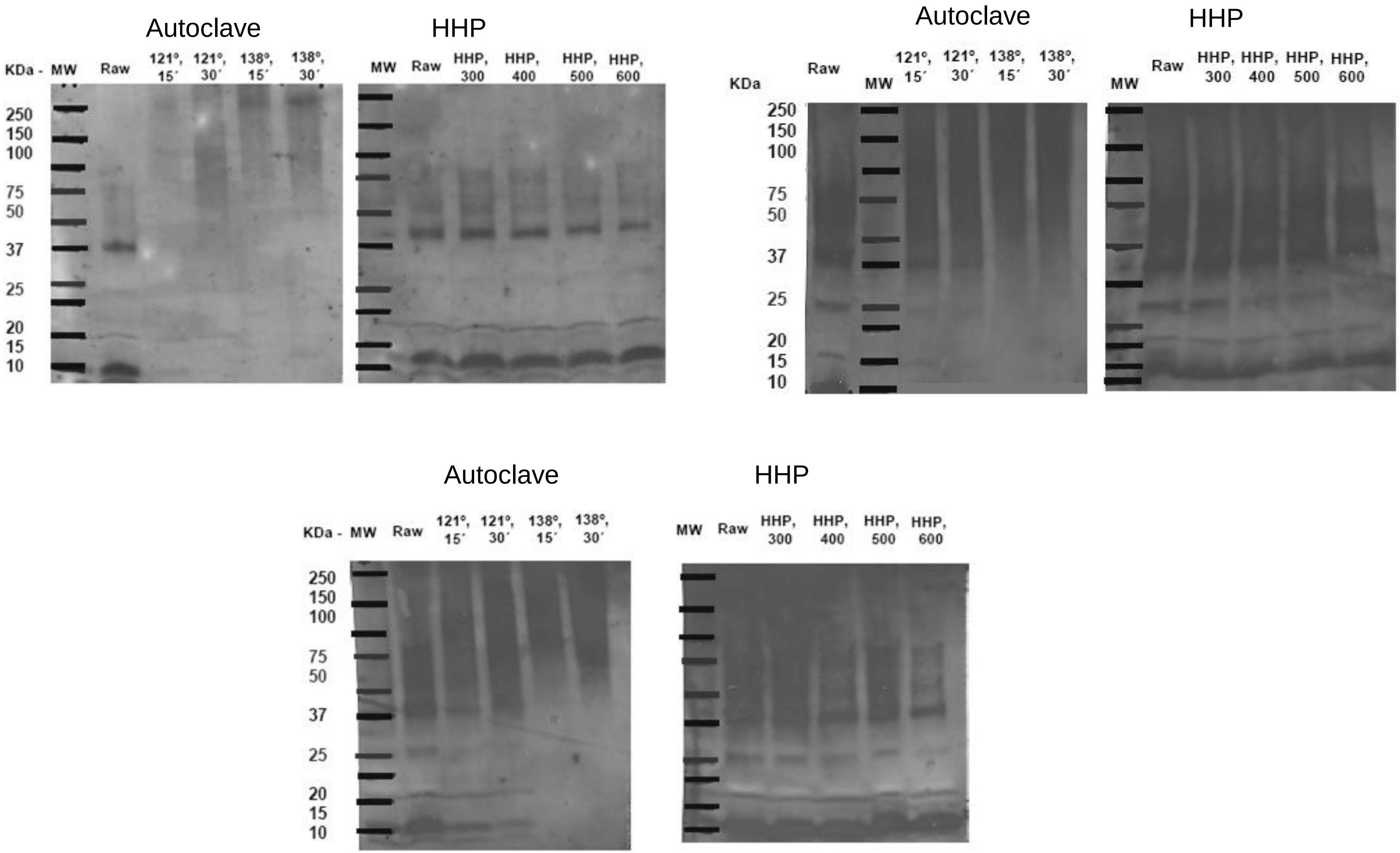

Figure S4. Western blot de almond (pool 4 patients)

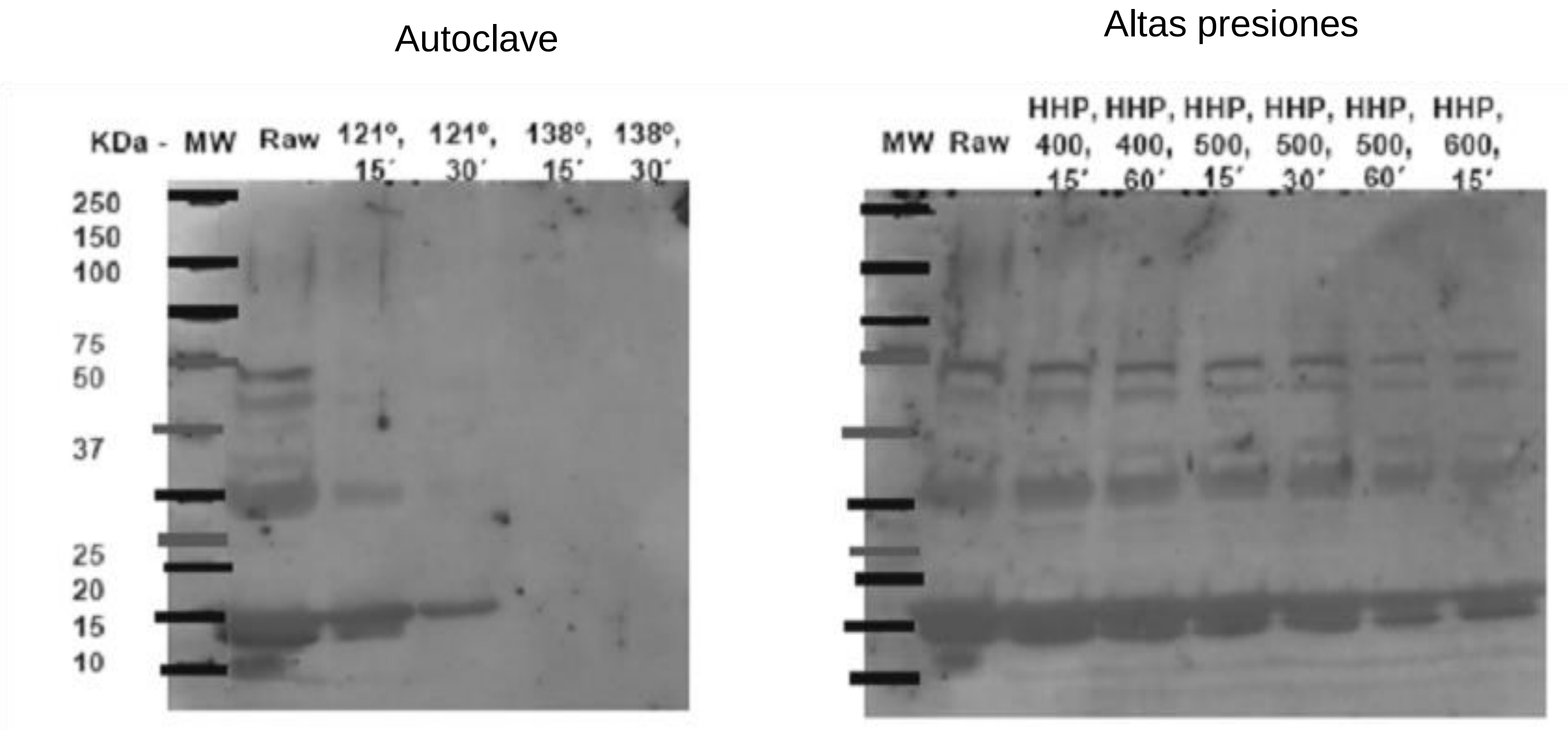

Figure S5. Immunodetection in Chesnut

SDS-PAGE 4-20%

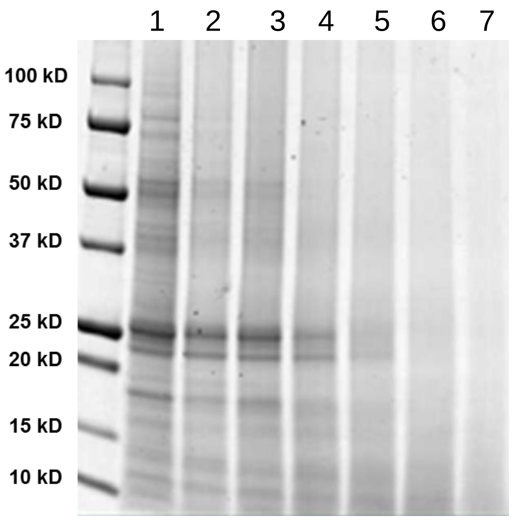

IgE WB

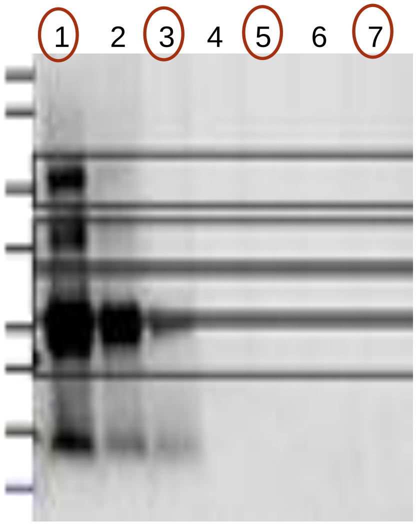

1- Untreated
2- Boiled 30 min
3- Boiled 60 min
4- Auto 121°C 15 min
5- Auto 121°C 30 min
6- Auto 138°C 15 min
7- Auto 138°C 30 min

IgE ELISA
